# World Health Organization Guidelines for Management of Acute Stress, PTSD, and Bereavement: Key Challenges on the Road Ahead

**DOI:** 10.1371/journal.pmed.1001769

**Published:** 2014-12-16

**Authors:** Wietse A. Tol, Corrado Barbui, Jonathan Bisson, Judith Cohen, Zeinab Hijazi, Lynne Jones, Joop T. V. M. de Jong, Nicola Magrini, Olayinka Omigbodun, Soraya Seedat, Derrick Silove, Renato Souza, Athula Sumathipala, Lakshmi Vijayakumar, Inka Weissbecker, Douglas Zatzick, Mark van Ommeren

**Affiliations:** 1 Department of Mental Health, Johns Hopkins Bloomberg School of Public Health, Baltimore, Maryland, United States of America; 2 WHO Collaborating Centre for Research and Training in Mental Health, University of Verona, Verona, Italy; 3 Institute of Psychological Medicine and Clinical Neurosciences, School of Medicine, Cardiff University, Cardiff, United Kingdom; 4 Department of Psychiatry, Allegheny General Hospital, Drexel University College of Medicine, Pittsburgh, Pennsylvania, United States of America; 5 International Medical Corps, Santa Monica, California, and Washington, D.C., United States of America; 6 FXB Center for Health and Human Rights, Harvard School of Public Health, Harvard University, Boston, Massachusetts, United States of America; 7 Amsterdam Institute for Social Science Research, University of Amsterdam, Amsterdam, the Netherlands; 8 WHO Collaborating Centre for Evidence-based Research Synthesis and Guideline Development, Bologna, Italy; 9 Department of Psychiatry, College of Medicine, University of Ibadan and University College Hospital, Ibadan, Nigeria; 10 Department of Psychiatry, Stellenbosch University, Tygerberg, South Africa; 11 School of Psychiatry and Ingham Institute, University of New South Wales, Sydney, Australia; 12 Institute of Psychiatry, Hospital das Clínicas, University of São Paulo Medical School, São Paulo, Brazil; 13 Research Institute for Primary Care and Health Sciences, Faculty of Health, Keele University, Keele, United Kingdom & Institute for Research and Development, Colombo, Sri Lanka; 14 SNEHA, Voluntary Health Services, Department of Psychiatry, Chennai, India; 15 Department of Psychiatry and Behavioral Sciences, University of Washington School of Medicine, Seattle, United States of America; 16 Department of Mental Health and Substance Abuse, World Health Organization, Geneva, Switzerland

## Abstract

Wietse Tol and colleagues discuss some of the key challenges for implementation of new WHO guidelines for stress-related mental health disorders in low- and middle-income countries.

*Please see later in the article for the Editors' Summary*

**Summary Points**

- The implementation of new WHO mental health guidelines for conditions and disorders specifically related to stress is likely to face obstacles, particularly in low- and middle-income countries.
- Formulation of evidence-based guidelines is complicated by limited knowledge regarding (a) the effectiveness of commonly implemented interventions, (b) the effectiveness of established evidence-based interventions when used in situations of ongoing adversity, and (c) the effectiveness of widely used cultural practices in LMICs. The application of the guidelines requires improved knowledge on how to reduce potentially harmful practices that are widely applied.
- The implementation of recommendations regarding psychotherapeutic interventions will require an approach that balances (a) strengthening the availability and capacity of specialists to train and supervise and (b) shifting to the delivery of psychotherapy by non-specialists.
- The strengthening of evidence for managing these conditions will require collaborative efforts by researchers and practitioners in a manner that is mindful of local sociocultural and health system realities.

## Why New Guidelines on Mental Health Conditions Specifically Related to Stress?

In 2009, the World Health Organization (WHO) launched the Mental Health Gap Action Programme (mhGAP), which has since been used in more than 50 countries worldwide. The mhGAP is aimed at improving access to evidence-based mental health interventions, by ensuring their integration within non-specialized (primary care) settings, the emphasis being on low- and middle-income countries (LMICs). The evidence-based guidelines formed the basis for the development of the mhGAP Intervention Guide [1].

Since then, WHO has been asked repeatedly to provide similar guidelines as a basis for an additional Intervention Guide Module, for conditions specifically associated with major stressors such as potentially traumatic events (e.g., involvement in severe accidents, armed conflicts, gender-based violence) and major losses (e.g., bereavement, displacement). Exposure to such major stressors is common in many LMICs [2].

The approach adopted by WHO was to ensure a comprehensive focus in developing guidelines for adults, children, and adolescents, comprising recommendations on pharmacological and psychological interventions. The guidelines include but extend beyond posttraumatic stress disorder (PTSD) to a range of conditions that are relevant to non-specialized health settings, including symptoms in the first month after exposure (acute traumatic stress symptoms, insomnia, enuresis, dissociation, and hyperventilation); PTSD; and bereavement in the absence of frank mental disorder. The development [3],[4] and content [5] of the evidence-based recommendations and resulting intervention module are described in more detail elsewhere. A brief summary of the recommendations is provided in Table 1.

**Table 1:** Overview of recommendations.

Mental health condition (broad)	Mental health condition (specific)	Recommendation	Strength of recommendation
Symptoms of acute stress (first month after exposure)^1^	Acute traumatic stress symptoms (intrusion, avoidance, hyperarousal) associated with significant impairment in daily functioning	CBT with a trauma focus (CBT-T) should be considered in adults	Standard^2^
		Benzodiazepines should not be offered to adults	Strong
		Antidepressants should not be offered to adults	Standard
		Benzodiazepines and antidepressants should not be offered to children and adolescents	Strong
	Insomnia	Relaxation techniques (e.g., progressive muscle relaxation or cultural equivalents) and advice about sleep hygiene (including advice about psychostimulants, such as coffee, nicotine, and alcohol) should be considered for adults	Standard
		Benzodiazepines should not be offered to adults	Standard
		Benzodiazepines should not be offered to children and adolescents	Strong
	Secondary nonorganic enuresis	Explanation of the negative effects of punitive responses, parenting skills training, and the use of simple behavioral interventions (i.e., star charts, toileting before sleep, and rewarding having nights without wetting the bed) should be considered	Strong
	Hyperventilation	Rebreathing into a paper bag should not be offered to children	Standard
PTSD		Individual or group CBT-T, EMDR or stress management should be considered for adults	Standard
		Individual or group CBT-T or EMDR should be considered for children and adolescents	Standard
		SSRIs and TCAs should not be offered as the first line of treatment for adults	Standard
		Antidepressants should not be offered to children and adolescents	Strong
Bereavement (without a mental disorder)		Structured psychological interventions should not be offered universally to (all) bereaved adults who do not meet the criteria for a mental disorder	Strong
		Structured psychological interventions should not be offered universally to (all) bereaved children and adolescents who do not meet the criteria for a mental disorder	Strong
		Benzodiazepines should not be offered to bereaved adults who do not meet criteria for a mental disorder	Strong
		Benzodiazepines should not be offered to bereaved children and adolescents who do not meet criteria for a mental disorder	Strong

1 For all symptoms of acute stress, a previous WHO GDG recommended Psychological First Aid as management strategy.

2 Strength of recommendations was evaluated in accordance with previous WHO mhGAP guidelines, which is based on GRADE (Grading of Recommendations Assessment, Development, and Evaluation) methodology (Barbui et al, 2010). A strong recommendation means that the guideline development group agreed that the quality of the evidence, combined with certainty about the values, preferences, benefits, and feasibility of this recommendation meant it should be followed in all or almost all circumstances. A standard recommendation means that there was less certainty about the combined quality of evidence and values, preferences, benefits, and feasibility of this recommendation; thus, there may be circumstances in which it will not apply.

SSRIs: selective serotonin reuptake inhibitors.

TCAs: tricyclic antidepressants.

Although these guidelines and companion intervention guide are an important first step, their success will rest on their actual implementation in settings with high needs for mental health care. In this paper, we discuss challenges encountered in the formulation of guidelines, as well as potential obstacles that may constrain effective implementation of these guidelines in low-resource settings. We also offer suggestions for how these obstacles may be overcome. The authors represent the Guideline Development Group (JB, JC, ZH, JTVMdJ, OO, SS, DS, RS, AS, LV, IW, DZ), WHO secretariat (MvO), and four consultants to the guideline development process (WAT, CB, LJ, NM).

## What Are the Key Obstacles on the Road Ahead?

First, the Guideline Development Group (GDG) (see author contributions) discovered that there is a dearth of scientifically rigorous research supporting many of the most commonly used interventions for managing conditions specifically associated with stress. To establish the evidence, the GDG identified recent systematic reviews, or they commissioned reviews in cases for which none were available. The evidence is particularly poor for children and adolescents: for three out of 11 questions asked, no specific recommendations could be made based on existing evidence. In relation to the absence of evidence in general, a notable example in adults concerns symptoms manifesting in the first month after exposure to major stressors. At the outset, the GDG commissioned evidence searches for a broader set of psychological interventions to manage acute traumatic stress symptoms in adults, including problem-solving counseling, relaxation, and psycho-education. However, it was deemed that there was insufficient evidence to recommend either in favor or against the use of these interventions. In humanitarian settings in LMICs specifically, earlier systematic reviews have shown that there is a wide gap between interventions that are commonly implemented and evidence for interventions in such settings [6].

Second, a key challenge is the limited availability of mental health resources in LMICs in general, creating a major obstacle to implementation of new mental health recommendations. Lack of resources takes a number of forms, including limits in basic mental health infrastructure, budget, and personnel, particularly in humanitarian settings [7]. Mental health is often a low priority for governments and donors, and too often there is a lack of political will to prioritize this area [8]. Where basic mental health resources do exist, there is a lack of specialized staff to provide the necessary training and supervision to ensure recommended psychotherapeutic interventions such as cognitive behavioral therapy (CBT) and eye movement desensitization and reprocessing (EMDR) can be implemented [9]. There is promising evidence, however, based on randomized controlled trials, that non-specialists (for example, community health workers or personnel without a formal mental health background working for non-governmental organizations) can successfully deliver psychotherapeutic interventions based on a task-sharing approach [10]–[12]. Nevertheless, it is uncertain whether psychotherapeutic interventions can be feasibly scaled up and sustained in naturalistic settings that lack the financial resources (e.g., for supervision) which were available to researchers when these interventions were tested.

Third, some practitioners may be reluctant to adhere to recommendations that caution against practices that are widely applied. These include, for example, recommendations not to offer benzodiazepines for acute traumatic stress symptoms, nor to offer structured psychological interventions for bereavement reactions in the absence of frank mental disorder. Studies have revealed the over-prescription of benzodiazepines in some LMIC health care settings [13],[14], supporting general impressions that in humanitarian settings the prescription of benzodiazepines for symptoms of acute stress (including insomnia) and bereavement is commonplace. Similarly, grief counseling is a popular intervention following bereavement in spite of the lack of evidence that it is necessary or effective [15]. Overall, there may be major challenges in achieving changes in practice in low-resource settings where the evidence-based alternative suggests more time-intensive management strategies or where expectations of help-seekers favor pharmacological management, as is common in many LMICs.

Fourth, mhGAP recommendations are based on evidence gathered mainly in well-resourced health settings in industrialized countries. There is uncertainty as to what extent the findings can be generalized across diverse sociocultural settings. Furthermore, there is a paucity of evidence concerning the effectiveness of (a) interventions for specific cultural idioms or concepts of distress [16] and (b) existing supportive cultural practices to manage stress-related conditions, such as yoga for stress management, or cultural mourning practices for bereavement. This is a paradox because international consensus guidelines for mental health and psychosocial interventions in humanitarian emergencies explicitly recommend identifying and building on such practices where possible [17], the evident advantages being accessibility, acceptability, and sustainability.

Fifth, a number of peer reviewers and the GDG raised questions about the specific challenges of providing effective treatments in contexts where stressors are ongoing. Situations of ongoing adversity, such as in the context of armed conflict, chronic poverty, or intimate partner violence, raise two important questions: (1) whether to prioritize social interventions over psychotherapeutic interventions in this population [18] and (2) if treatments are equally effective and safe for those exposed to ongoing major stressors. Limited knowledge is available to guide decisions on both issues. With regard to the first question, consensus guidelines have recommended addressing social and psychological issues simultaneously in a multilayered, multisectoral approach. However, randomized trials have not yet indicated whether this is more effective than single-intervention approaches. With regard to the second question, there is some evidence that treatments can be effective in situations of ongoing adversity [19],[20], whereas other studies have shown reduced treatment benefits for populations facing chronic adversity [21].

## How Can These Obstacles Be Overcome?

First, concerted action is needed to strengthen the evidence base for interventions. This will require testing the efficacy of treatments for conditions specifically related to stress that previously have proven efficacious, generally in high-income countries. Also, it will require determining the efficacy of interventions that are currently very popular in practice but have not been rigorously studied. Currently popular interventions include non-specific counseling, psycho-education, structured recreational and sports activities, and provision of child-friendly spaces. In relation to the current knowledge base, there are both consistent and inconsistent findings [22]. For example, amongst children and adolescents with PTSD, CBT has been shown to be more effective than supportive counseling for a range of outcomes [23]. In humanitarian settings, some studies have shown benefits of counseling [24],[25], whereas others have not [26]–[28]. It is challenging to make broad conclusions based on existing studies given the heterogeneity of counseling approaches applied, and further rigorous research is required. Child-friendly spaces provide an additional example. This popular intervention aims at promoting and supporting resilience and well-being amongst children and young people who have recently experienced natural or human-made disasters by provision of community-organized, structured activities conducted in a safe, child-friendly, and stimulating environment. A recent systematic review found no randomized controlled trials evaluating this methodology, and only one study applying a comparison group with pre- and post-intervention measurement of outcomes [29].

The evidence base could benefit from action on two fronts: supporting research efforts in LMICs, as well as building the capacity of agencies to measure the outcomes of interventions in the course of program implementation. Research capacity building in LMICs could assist in initiating research projects that are relevant to low-resource settings, e.g., testing culturally congruent approaches for stress management. A recent initiative to set research priorities for mental health and psychosocial support in humanitarian settings, in which there was strong representation from personnel working in LMICs, produced a largely practice-informed research agenda; notably, there was little focus on topics that have dominated debates in the peer-reviewed literature. For example, the agenda focused on a range of disorders and conditions besides PTSD, in spite of the latter diagnosis being the subject of most attention in the literature [30]. Building research capacity within agencies implementing interventions (e.g., government and non-governmental organizations) has the potential to improve knowledge on a larger scale and in a shorter time frame. For example, including outcome indicators measuring mental health as a standard monitoring practice could assist in rapidly identifying which practice elements are associated with positive changes. Finally, future research efforts should take costs of treatments into account to better inform policy and practice.

Second, the limited availability of mental health resources in LMICs will need to be addressed for successful implementation of these guidelines. For example, building the capacity to train and supervise psychotherapeutic interventions in non-specialist settings will be crucial to ensure that such services are sustainable and of sufficient quality [31]. This is likely to require a balanced approach in building capacity of both specialist supervisors and non-specialist health workers in community and primary health care settings [32]. Future studies could explore implementing and evaluating stepped care models in which (a) those who are in greatest need receive timely interventions, (b) people are identified and receive interventions initially from community workers, and (c) those people are referred to progressively more resource-intensive interventions, depending on their response to first-line treatments [33]. In addition, a promising direction concerns transdiagnostic approaches in which mental health practitioners learn skills across a variety of therapies to address a range of common mental disorders [34]. This approach seems most desirable given the high levels of comorbidity (for example, involving PTSD, anxiety, and depression) in the context of major stressors, and the lack of feasibility of training personnel in numerous separate interventions that address single conditions.

Third, if the aim is to ensure that negative recommendations are implemented (that is, to discontinue established practices) it is important to understand (a) the reasons behind practitioners' current use of contra-indicated interventions, (b) barriers to using evidence-based approaches, and (c) strategies that will encourage practitioners to take up evidence-based alternatives. Such research falls within the scope of dissemination and implementation science, a field of research that has not yet received sufficient attention for mental health interventions in LMICs [35]. For example, important research questions in this regard may include the following: What are the most important determinants of currently employed pharmacological and non-pharmacological interventions (e.g., demand-side preferences of clients versus delivery-side training and preferences of mental health workers)? What are the perspectives of mental health professionals in LMICs with regard to evidence-based approaches that often originate in high-income countries? What are key barriers and facilitators for implementation and scaling up of evidence-based interventions? What types of adaptations are necessary for evidence-based interventions to be compelling and practical from a contextual and cultural perspective? The limited available research on uptake of guidelines by practitioners has shown that it is critical to fit guidelines to local contexts, to make recommendations sensitive to how work is organized locally, and to ensure feedback mechanisms to monitor implementation of recommendations [36],[37].

Fourth, lack of knowledge with regard to cultural concepts of distress and the effectiveness of existing cultural practices need to be addressed. This will require a willingness to take seriously local perspectives on mental health across highly diverse sociocultural settings in LMICs. Such efforts would benefit from multidisciplinary collaboration, in which experts in qualitative research (e.g., to identify the phenomenology of cultural concepts of distress, views on determinants of these concepts, and help-seeking patterns) join hands with experts in quantitative research (e.g., to identify prevalence rates and establish efficacy of interventions). A recent meta-analysis found a particularly high level of overlap between locally defined cultural concepts of distress and a diagnosis of PTSD. However, one of the major shortcomings of this literature concerned the failure to include cultural concepts of distress in treatment outcome studies [16].

Finally, implementation efforts need to be more responsive to the reality that populations affected by humanitarian crises in LMICs commonly are living in settings of ongoing exposure to major stressors. Further research needs to confirm whether evidence-based treatments can be safely and sustainably implemented in low-resource settings with populations exposed to high levels of ongoing adversity and to identify additional promising intervention approaches. Clinical interventions are only part of the picture, given the impact of social and economic factors on mental health in these settings. There is a need to strengthen preventive approaches that address the major social determinants of mental health, including poverty, gender-based violence, and social exclusion [38]. One approach would be to combine preventive and treatment interventions—in a way, to simultaneously target the causes and consequences of adversity, thereby addressing both ends of a vicious cycle. For example, an intervention that would relieve symptoms of mental disorders in women exposed to past intimate partner violence could, at the same time, aim at empowerment of women to prevent future violence, engagement of men, and awareness-raising in the community as a whole about the deleterious effects of domestic violence. Previous research has indicated that psychotherapeutic treatments by themselves may lower chances for future victimization [39].

In conclusion, although the new WHO recommendations on conditions specifically related to stress present an important step forward, there will be many challenges in implementation. The road ahead is to address these challenges through concerted and multidisciplinary efforts by policy makers, practitioners, and researchers.

## Disclaimer

The authors are responsible for the views expressed in this article and, except for the specifically noted recommendations, they do not necessarily represent the decisions, policies, or views of WHO.
